# Design and methodology of the *A*ging *Ne*phropathy *S*tudy (AGNES): a prospective cohort study of elderly patients with chronic kidney disease

**DOI:** 10.1186/s12882-020-02116-w

**Published:** 2020-11-07

**Authors:** Venceslau A. Coelho, Giovani GN. Santos, Carla M. Avesani, Cicero Italo L. Bezerra, Luana Cristina A. Silva, Julia C. Lauar, Bengt Lindholm, Peter Stenvinkel, Wilson Jacob-Filho, Irene L. Noronha, Roberto Zatz, Rosa M. A. Moysés, Rosilene M. Elias

**Affiliations:** 1 LIM 66, Division of Geriatrics, Faculdade de Medicina Universidade de Sao Paulo, Sao Paulo, Brazil; 2 Division of Geriatrics, Hospital das Clinicas HCFMUSP, Sao Paulo, Brazil; 3 Division of Renal Medicine and Baxter Novum, Department of Clinical Science, Intervention and Technology, Karolinska Institutet, Stockholm, Sweden; 4 Division of Nephrology, Hospital das Clinicas HCFMUSP, Sao Paulo, Brazil; 5 LIM 29, Division of Nephrology, Faculdade de Medicina Universidade de Sao Paulo, São Paulo, Brazil; 6 LIM 16, Division of Nephrology, Faculdade de Medicina Universidade de Sao Paulo, São Paulo, Brazil

**Keywords:** End stage renal disease, Dialysis, Mortality, Renal replacement therapy, Fragility, Sleep, Cognition

## Abstract

**Background:**

Renal replacement therapy (RRT) is usually indicated for patients with chronic kidney disease (CKD) with glomerular filtration rate below 10 ml/ml/min/1.73m^2^. However, the need for RRT and timing of dialysis initiation are debatable for patients aged 70 years or older. We here describe the study design and methodology of the Aging Nephropathy Study (AGNES) protocol that aims at evaluating to what extent geriatric-related conditions such as frailty, cognitive dysfunction, and presence of comorbidities have an impact on survival and RRT initiation in this group of patients. In this manuscript we provide detailed information about the AGNES study design and methodology.

**Methods:**

AGNES is a prospective observational cohort that aim to investigate clinical, biochemical and demographic factors associated with RRT initiation and mortality of patients with CKD stage 4 or 5 who are aged 70 years and older. We plan to include 200 patients over 5 years. Clinically stable outpatients on conservative management for at least 6 months will be recruited from the Nephrogeriatric Clinic at the *Hospital das Clinicas da Universidade de Sao Paulo*, Brazil. Eligible patients are submitted to a full clinical examination, geriatric assessment, and blood test at baseline. Following the baseline visit the patients are being monitored during an observational follow up period of at least 12 months during which patients will be contacted in the clinic at their regular follow up or by phone until either RRT initiation or death occurs. This cohort includes evaluation of cognition by the education-adjusted 10-point Cognitive Screener (10-CS), frailty by Fried index score, a complete nutritional assessment (by body composition assessment, global subjective assessment and dietary intake), comorbidities by Charlson comorbidity index and biochemical markers including FGF-23 and Klotho.

**Discussion:**

The AGNES cohort, a real-world study of current clinical practice in elderly patients with advanced CKD prior to dialysis initiation, will shed light into progression of CKD and its complications, indications of RRT and factors determining survival. This investigation will elucidate to what extent geriatric conditions, nutritional status and clinical factors are associated with survival, quality of life and RRT initiation in elderly CKD patients not yet on dialysis.

**Trial registration:**

Registered on ClinicalTrials.gov on 18 October 2019 (NCT04132492).

## Background

Chronic kidney disease (CKD) is a common clinical condition in elderly individuals and is associated with large morbidity and mortality. The high prevalence of hypertension and diabetes in the aging population partially explains the high burden of CKD in the elderly population because these two conditions are among the most common causes of CKD.

Considering the KDIGO classification of CKD, the overall prevalence of patients with CKD stage 5 CKD (estimated glomerular filtration rate - eGFR < 15 ml/ml/min/1.73m^2^) is much higher in elderly individuals [1]. Assessment of eGFR is now a common practice that has replaced creatinine clearance measurements in the clinical practice; however, it is not clear which is the best equation for eGFR calculation in elderly patients especially in those with advanced CKD. Whereas the CKD-EPI creatinine-based eGFR overestimates the true GFR in this population, eGFR calculated by the CKD-EPI cystatin C formula is described to be better for older individuals, and the equation derived from the Berlin initiative study (BIS-1) also seems to be reliable for use in stages 1–3 CKD. Recently, a study tested these equations against the gold-standard inulin clearance and concluded that none of them was superior in estimating GFR in elderly individuals [2].

Managing CKD in the elderly is challenging and it is not always clear if dialysis initiation will provide a survival or health benefit. Thus, the best approach considering the choice of conservative care vs. renal replacement therapy (RRT) is still debatable. This is mainly because there is still limited information about these issues and specific evidence-based guidelines for elderly patients with CKD are lacking. However it is evident that geriatric issues such as frailty, cognition, and treatment complications must be taken into account in the shared decision making process when choosing between conservative treatment and RRT. There is a general belief that the *relative risk of mortality* associated with low eGFR is smaller in elderly patients than in younger individuals. However, elderly patients starting on dialysis has a very high *absolute risk of death* that associates with baseline diseases, sleep disorders, and deteriorated functional status and other complications. Indeed, a recent study showed that the mortality rate among patients 65 years and older was 22.5% at 30 days after starting dialysis, 44.2% at 180 days, and 54.5% at 365 days [3]. In addition, factors affecting life quality such as functional status may deteriorate as well. In a study based on a national registry of patients undergoing dialysis in the United States of America, initiation of dialysis among nursing home residents with an average age of 73 years old was associated with a substantial and sustained decline in functional status [4].

Considering the above mentioned, this study will enrol elderly patients (70 years and older) with stage 4 and 5 CKD under conservative management as an attempt to identify clinical, demographic and biochemical factors associated with mortality and the initiation of RRT, considering the presence of comorbidities and geriatric conditions.

## Methods

### Primary hypotheses

Our primary hypothesis is that mortality is associated with greater number of comorbidities and with a high impact of geriatric issues. The decision of beginning RRT will be more likely to occur in patients with fewer burdens of geriatric syndromes.

### Exploratory hypotheses

- Frailty and cognition deficit will be associated with high mortality rate;
- Presence of comorbidities, in particular diabetes and congestive cardiac failure will be associated with mortality and beginning of RRT;
- Klotho and Fibroblast growth factor (FGF-23) will be associated with both outcomes, mortality and beginning of RRT;
- Protein energy wasting and sarcopenia nutritional status will be associated with high mortality rate;
- Sleep disorders, particularly poor sleep quality, will be associated with mortality.

### General study design

AGNES was designed as a prospective unblinded cohort from a single academic center, evaluating risk factors of mortality and biomarkers associated with beginning of RRT. Patients who agree to participate are signing the written informed consent. Baseline data are collected in the same day.

### Ethical consideration

The AGNES was registered at clinicaltrials.gov (#NCT04132492). It is conducted in accordance with the Declaration of Helsinki. The Local Board Ethical Committee has approved the protocol (*Comissão de Ética para Análise de Projetos de Pesquisa do HCFMUSP*, application 97,812,918.3.0000.0068, number 3.438.278). Patients are providing written informed consent before their enrollment in the study. In order to maintain confidentiality, the investigators deidentified the health information.

### Setting

Patients are being recruited from the Nephrogeriatric outpatient clinic at the *Hospital das Clinicas, Universidade de Sao Paulo*, Brazil, which adopts a multidisciplinary approach and team. The team includes dietitian, nurse, nephrologist, and geriatric physician.

#### Inclusion criteria

Patients aged 70 years old or older, both sexes, with CKD stage 4 or 5 according to eGFR calculated by the CKD-EPI formula;

#### Exclusion criterion

Patients with life expectancy lower than 6 months according to the investigator’s judgment; patients with untreatable cancer.

### Study visit procedures

Potential candidates for the AGNES study are being invited by the investigator and screened for eligibility. Eligible patients are being approached for recruitment. After review of study procedures, risks and benefits, those who agreed and signed the informed consent form are included. Blood sample is collected in the same visit to biorepository.

Baseline parameters recorded include: global geriatric assessment (GGA) performed with the supervision of the geriatric physician, demographic and clinical history, medications in use, nutritional assessment, questionnaires for sleep disorders, non-invasive hemodynamic monitoring and body composition assessment by bioelectrical impedance (BIA).

### Global geriatric assessment (GGA)

All participants are being submitted to a GGA at baseline, performed by the same physician. GGA includes a structured interview, physical examination, and a geriatric functional assessment. Geriatric domains assessed are functional status (daily life activities), nutritional status, physical health, cognition, depression, and social and family support [5].

The Katz Index of Independence in Activities of Daily Living (ADL) [6], validated to the Portuguese language [7] will be used to assess the functional status. The Katz index of ADL provides measures independence and need of assistance during bathing, dressing, toileting, transferring, continence, and feeding. Higher scores indicate more patient independence.

The Geriatric Depression Scale (GDS)-Short Form will be used to identify presence of depression and its severity [8]. This test yield scores that range from 0 to 15 with higher scores indicating higher depression probability.

The education-adjusted 10-point Cognitive Screener (10-CS) [9] assesses cognitive performance. Individuals with scores ≥8 are considered to have normal or near-normal cognition.

### Frailty index

Frailty is being evaluated using criteria established by Fried et al. [10], with 5 components as following:

1. Unintentional weight loss ≥5% of body mass in the last year;
2. Weakness (loss of palmar prehension force), adjusted for age and body mass index (BMI).
3. Exhaustion (audited information based on two questions from Center for Epidemiological Studies Depression (CES-D) scale; a score from 1 [fatigue or exhaustion felt rarely or not at all] to 4 [fatigue or exhaustion felt most of the time], 3 or 4 points means that the test is positive for decreased physical activity);
4. Slow gait speed (walking time over a distance of 4.6 m); interpretation of results takes into account sex and height;
5. Low physical activity (measured by weekly energy expenditure in kcal, based on self-report of specific physical activities and exercises performed according to validated questionnaires such as the IPAC - International Physical Activity Questionnaire validated to Portuguese)

Patients who fulfilled none of the criteria are considered non-fragile, patients who fulfilled 1 and 2 criteria are classified as pre-fragile, and patients who fulfilled 3 or more criteria are classified as fragile.

### Follow up

After a baseline visit, patients will be followed by telephone contact every 4 months to check mortality and the beginning of RRT. The follow up will last at least 12 months. In addition, patients will be routinely followed in the same outpatient clinic with an average interval between visits of 4 to 5 months. Patients can withdraw their consent to participate in the study at any time.

### Nutritional assessment

The nutritional assessment includes anthropometric measurements, body composition by BIA, handgrip strength (HGS) by a handgrip dynamometer, 7-point subjective global assessment (7p-SGA) and the dietary intake by 24-h food recall.

The BMI is calculated by dividing weight (kg) by the square of the height (m^2^). BMI (kg/m^2^) is classified according to the cutoffs proposed by the Pan American Health Organization criteria for the elderly [11] as underweight (BMI < 23 kg/m^2^), normal body weight (BMI ≥ 23 and < 28 kg/m^2^), overweight (BMI ≥ 28 and < 30 kg/m^2^), and obese (BMI ≥ 30 kg/m^2^). BMI is being also classified according to the conventional Worth Health Organization as underweight (BMI < 18.5 kg/m^2^), normal body weight (BMI ≥ 18.5 and < 25 kg/m^2^), overweight (BMI > 25 and < 30 kg/m^2^), and obese (BMI ≥ 30 kg/m^2^). Anthropometric parameters are assessed with patients in the standing position and by the same observer. The following measurements are evaluated in the right side of the body using either a non-stretchable tape or a skinfold caliper (Cescorf® Scientific model, Cescorf Equipaments, Brazil) as appropriate: calf circumference (CF), right mid-upper arm circumference (MUAC) and triceps, biceps, triceps, subscapular and suprailiac skinfold thickness. An average of 3 consecutive measurements are considered for each site assessed. The body fat percentage is calculated as proposed by Durnin & Wormersley [12].

The HGS evaluates strength and muscle quality [13], using a handgrip dynamometer (E-clear®, model EH 101, Cei Technology, Taoyuan City, Taiwan). With the arms along the body, the patients are encouraged to press the tool with maximum strength in response to a voice command, as recommended by the American Society of Hand Therapists. The maximum contraction of the dominant arm for 3 s is evaluated. The measurements are repeated 3 times, and the highest value is considered and expressed in kg (and as percentage of controls with same age and sex).

The Subjective Global Assessment (SGA) [14], a well-validated tool to assess nutritional status, is being applied to all participants, in a version of 7 points, according to the CANUSA study [15]. The 7p-SGA involves the assessment of medical history: changes of body weight and dietary intake, gastrointestinal symptoms, and functional capacity. In addition, a physical examination of body fat reserves, muscle mass and presence of edema is also evaluated. Each component has a score from 1 to 7. The 7p-SGA scale [16], validated to Portuguese [17] classifies patients as well-nourished (score 7 to 6), mild to moderate protein-energy wasting (score 5 to 3) and severe protein-energy wasting (score 2 to 1) [16].

Phase angle, intra- and extracellular water, total body water, fat mass, fat free mass, and skeletal muscle mass are measured by the single multifrequency BIA device InBody™ S10 (Biospace Co., Ltd., Korea) with the software from the equipment. The patient is placed in the supine position, with arms and legs separated from the body. Any metal object is removed. Eight electrodes are positioned, two in each limb, according to manufacturer instructions.

In total, the nutritional assessment lasts about 30 min in each patient in addition to the 7p-SGA, which lasts for another 10 min.

The dietary intake of all patients is assessed through a 24-h dietary recall interview (R24h). The R24h consists of defining and quantifying all foods and beverages ingested in the pre-interview period, which may be in the preceding 24 h or, more commonly, in the day previous to the assessment [18]. Quantitative assessment of intake is being done using DietBox nutritional calculation software and diet quality is assessed using the Diet Quality Index, as previously described [19].

Sarcopenia is being evaluated by the SARC-F, a self-reported questionnaire [20]. This tool has 5 components related to functional status: strength, assistance with walking, rising from a chair, climbing stairs, and falls. Each component scores from 0 to 2, with higher scores suggesting sarcopenia.

### Charlson comorbidity index (CCI)

The Charlson comorbidity index [21] is a widely used method predicting mortality by weighting comorbidities, validated in the renal disease context [22]. The CCI contains 19 issues including diabetes, hemiplegia, renal disease, congestive heart failure peripheral vascular disease, chronic pulmonary disease, mild and severe liver disease, leukaemia, lymphoma, metastatic tumor, and acquired immunodeficiency syndrome, each of which was weighted according to their potential influence on mortality.

### Non-invasive hemodynamic monitoring

Non-invasive hemodynamic monitoring is evaluated using the Finometer® device (Finapress Medical System B.V., Amsterdam, The Netherlands) [23], which is a low-risk device able to measure beat-to-beat continuous blood pressure [24]. All patient’s measurements is being taken in the supine position, after a rest period of 10 min. The Finometer is calibrated for at least 2 min as recommended by the manufacturer [23]. All measurements are conducted in a quiet room, between 7 and 10 am. Blood pressure is measured in the nondominant arm and recorded for 7 min. The finger cuff is placed on the middle finger. The following parameters are evaluated: systolic, diastolic and mean blood pressure, systolic volume, heart rate, stroke volume, cardiac output, and total systemic peripheral resistance. Active data will be exported with the BeatScope Easy software (MedTech Inc., Burlington, Ontario, Canada), according to the BeatScope 5-s averages method.

### Pittsburgh sleep quality index (PSQI)

The PSQI is an instrument used to measure the quality and patterns of sleep in adults [25], validated to the Portuguese Language [26]. Seven domains are evaluated: subjective sleep quality, sleep latency, sleep duration, sleep efficiency, sleep disturbances, use of sleep medication, and daytime dysfunction over the last month. Patients are classified as poor sleepers or good sleepers when the sum of scores is > or < 5, respectively.

### Subjective sleepiness

The Epworth Sleepiness Scale (ESS) assesses subjective daytime sleepiness, which is a self-administered questionnaire [27], validated to the Portuguese Language [28]. Scores rate on a scale of 0 to 3 how likely they are to fall asleep in 8 common situations. Higher scores indicate more sleepiness.

### Laboratory biomarkers

About 8–10 mL of blood specimens are collected in two different tubes (serum and plasma) at the baseline visit and saved in a box with ice. Specimens are centrifuged, identified and stored in the biorepository at the temperature of − 80° Celsius, in the Investigation Laboratory 16 (LIM-16) of the *Universidade de Sao Paulo*, Sao Paulo, Brazil. Biomarkers of bone and mineral metabolism such as FGF-23 and Klotho will be measured at the end of the study period.

Standard laboratory measurements such as urea, creatinine, estimated glomerular filtration rate (eGFR), hemoglobin, calcium, phosphate, bicarbonate, serum albumin, 25(OH) vitamin D and parathyroid hormone (PTH) will be collected from the patient’s charts.

### Power analysis

The sample calculation to obtain *p* < 0.05, with a cross-sectional design, and 80% of beta error, was calculated to require 197 individuals or 119 events. Based on the average monthly attendance of patients with the inclusion profile in the study and considering refusal and loss of follow-up in up to 20% of the sample, we plan to include this sample in 4.5–5 years.

### Statistical analysis

First, we will perform a descriptive analysis of the demographic, nutritional and clinical parameters of the included patients. Specific data includes geriatric syndromes, full nutritional assessments, muscle strength (based on HGS), body composition, sleep questionnaires, CCI, and non-invasive hemodynamic parameters. Continuous data will be presented as mean ± SD and categorical data will be presented as proportion. Normality distribution will be tested by D’Agostino omnibus test. Patients will be classified according to clinical evaluation according to the cognitive deficit, frailty, nutrition status, stage of renal function (4 or 5) and the presence of sleepiness and poor quality of sleep. Exploratory analyses will include an association between any of the above-mentioned clinical diagnoses with the 2 outcomes: death or beginning of renal replacement therapy. These outcomes will be closely monitored by the study team in the routine visits or and/or by telephone contact. A *p* value < 0.05 will be considered significant. Analyses will be performed using SPSS 22.0 (SPSS Inc., Chicago, IL, USA) and *GraphPad Prism® software* (GraphPad Software, Inc., CA, USA).

## Discussion

We have initiated the recruitment of elderly patients with CKD stages 4 and 5 under treatment at the multidisciplinary outpatient clinic. This study will bring novel data on risk factors in the context of CKD in individuals with comorbidities and geriatric syndromes, a scenario that raises many unanswered questions about when and to whom indicate RRT. This cohort will allow for the assessment of clinical factors that potentially impact the risk of death. Furthermore, this study aims to characterize the clinical and demographic profiles of elderly patients with CKD not receiving dialysis. Ultimately, this study will help elucidating if the risk of mortality will be higher than the risk of not starting dialysis in this population and the factors independently associated with each one of these outcomes.

This study includes several strengths such as a multidisciplinary approach that brings clinical information beyond nephrology since geriatric syndromes and nutritional status will be assessed. To the knowledge of the authors, this is the first study on Latin America with this profile.

Elderly patients with advanced CKD faces a high risk for common geriatric conditions such as cognition deficit [29]. It is known that cognitive function broadly deteriorates as CKD progresses from stage 1 to stage 5. As CKD progresses, deficits in executive functioning, verbal fluency, logical memory, orientation, and concentration become more evident. People with end-stage kidney disease manifest significant cognitive deficits, along with delayed and immediate memory, visuospatial impairment, and overall cognitive impairment [30]. Cognition, however, does not seem to increase the risk for CKD progression [31]. In addition, it has been demonstrated that cognition declines faster in dialysis patients compared with CKD patients receiving conservative treatment, and in hemodialysis patients compared with peritoneal dialysis patients [32].

Frailty and poor functional status are risk factors for adverse patient outcomes that may be useful additions to prognostic tools in patients with CKD [33, 34]. Recent evidence supports the prognostic significance of frailty for functional decline and poor health outcomes in patients with CKD [35]. Frailty is also associated with bone loss among patients with CKD [36]. Therefore, our study will add body to the literature showing if frailty will predict death or dialysis initiation in elderly patients with CKD.

The transmembrane form of α-Klotho serves as a co-receptor for the fibroblast growth factor (FGF)23 and may have independent homeostatic functions as a hormone [37]. In patients with CKD, there is a disruption of the FGF23-αKlotho axis manifesting in deficient klotho expression and FGF23 excess. Multiple lines of evidence show that the anti-aging and cognition-enhancing protein Klotho fosters neuronal survival, increases the anti-oxidative stress defense, and promotes remyelination of demyelinated axons [38]. Klotho and FGF-23 have been already associated with cardiovascular disease mortality in patients with CKD [39]. A randomized Italian trial showed that higher plasma klotho concentration was associated with lower likelihoods of frailty in individuals aged 65 years or over [40]. This finding highlights the importance of Klotho measurement in elderly patients with CKD.

Sarcopenia has been associated with mortality in patients with CKD in both conservative management [41] and dialysis [42]. Nutritional assessment in the current study will provide valuable information that might help to understand risk factors for death and the beginning of renal replacement therapy. In addition, we will be able to understand dietary patterns in the elderly population with CKD, and analyze associations of nutritional status with functional status, frailty and mortality.

Sleep disorders are common among patients with CKD, who experience both poor sleep quality and a lower amount of sleep when compared to the general population [43]. Indeed, sleep duration has been associated with progression to end-stage renal disease [44]. However, most studies were confined to dialysis patients and there is scarce information on sleep disorders among CKD patients within the conservative management scenario. Since sleep disorders are highly prevalent in patients with CKD, and has been associated with fluid overload [45] and mineral and bone metabolism [46] and higher age elderly individuals present, naturally, a reduction of sleep duration, the current study might contribute to the body of the literature in this field.

The results and interpretation of this study should consider some limitations: first, as this is not a large multicenter study the number of patients is relatively know. Second, polysomnography will be available only for a subset of patients. Third, the choice of starting renal replacement therapy is a shared-decision and many factors not investigated within the study such as family and social support may bias this outcome. However, we aim for a real-world study that likely will mimic the usual clinical practice, and considering that it is unethical to randomize patients to start dialysis or not regardless of their wishes, the current design is the one achievable.

In summary, this clinical study intends to address a common challenging situation in renal care of elderly individuals with CKD namely the choice between initiating renal replacement therapy vs. continue with conservative management. One of many questions we hope to answer is: Who are at higher risk of death when initiating dialysis in elderly patients and in whom would conservative management be more appropriate? However, we will evaluate not only survival benefits but also patient-reported outcomes including parameters related to health benefits and quality of life. The identification of features associated with patient survival, health benefits and quality of life may inform treatment strategies for better management of elderly patients with CKD. Ultimately, this study will address what, for whom and when considering a given treatment for individuals of the same age but with different functional state will result in opposite outcome.

## Data Availability

The datasets are being generated since the study is ongoing and are not publicly available but are available from the corresponding author on reasonable request.

## Abbreviations

7p-SGA: 7-point subjective global assessment
10-CS: 10-point Cognitive Screener
ADL: Activities of Daily Living
AGNES: Aging Nephropathy Study
BIA: Bioelectrical impedance
BIS-1: Berlin initiative study
BMI: Body mass index
CCI: Charlson Comorbidity Index
CF: Calf circumference
CES-D: Center for Epidemiological Studies Depression
CKD: Chronic Kidney Disease
ESS: Epworth Sleepiness Scale
eGFR: Estimated glomerular filtration rate
GDS: Geriatric Depression Scale
GGA: Global geriatric assessment
FGF-23: Fibroblast growth factor 23
HGS: Handgrip strength
IPAC: International Physical Activity
MUAC: Right mid-upper arm circumference
PTH: Parathyroid hormone
PSQI: Pittsburgh Sleep Quality Index
RRT: Renal replacement therapy
